# Supplementary figures and images for: Modulation of hepatitis B virus pregenomic RNA stability and splicing by histone deacetylase 5 enhances viral biosynthesis
Source: PLoS Pathog. 2020 Aug 21;16(8):e1008802. doi: 10.1371/journal.ppat.1008802 (PMC7467325; doi:10.1371/journal.ppat.1008802)

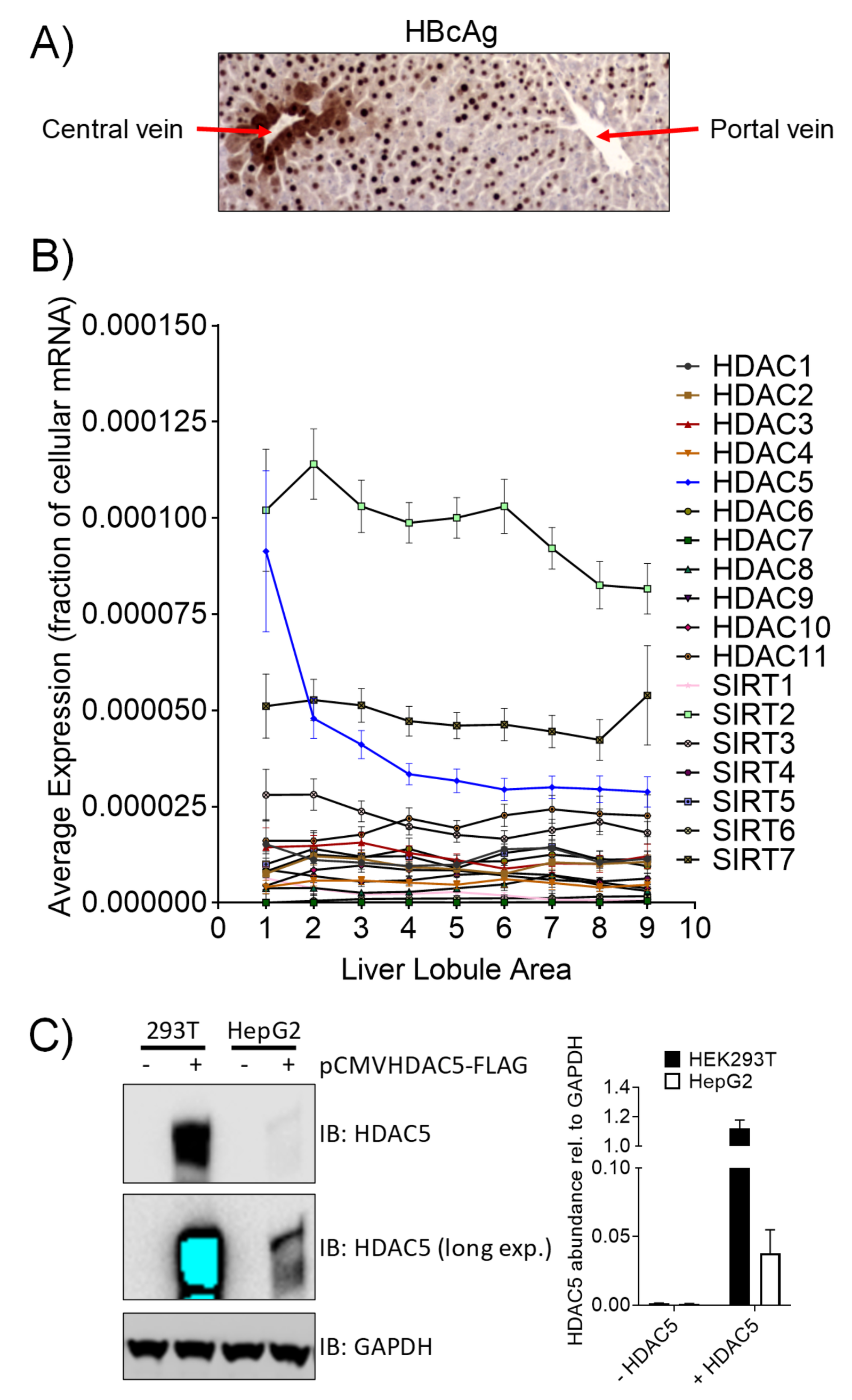

Supplement: S1 Fig — (A) Immunohistochemical staining of an HBV transgenic mouse liver showing the presence of nuclear HBcAg throughout the liver whereas cytoplasmic staining is located primarily in the centrolobular hepatocytes (adapted from [34]). (B) Single-cell RNAseq analysis of mouse liver tissue for HDACs and sirtuins (SIRTs) demonstrating that HDAC5 is highly expressed in the liver and exhibits zonation towards the pericentral area of the liver lobule (adapted from [33]). (C) Western blot analysis of HDAC5 expression in HEK293T and HepG2 cells. Cells were transfected with the HDAC5 expression vector, pCMVHDAC5, and whole cell lysates were analyzed for HDAC5 expression after 72 hours using the mouse anti-human HDAC5 monoclonal antibody (C-11, Santa Cruz). The level of HDAC5 expression relative to GAPDH is presented. (TIF) [file ppat.1008802.s001.tif]

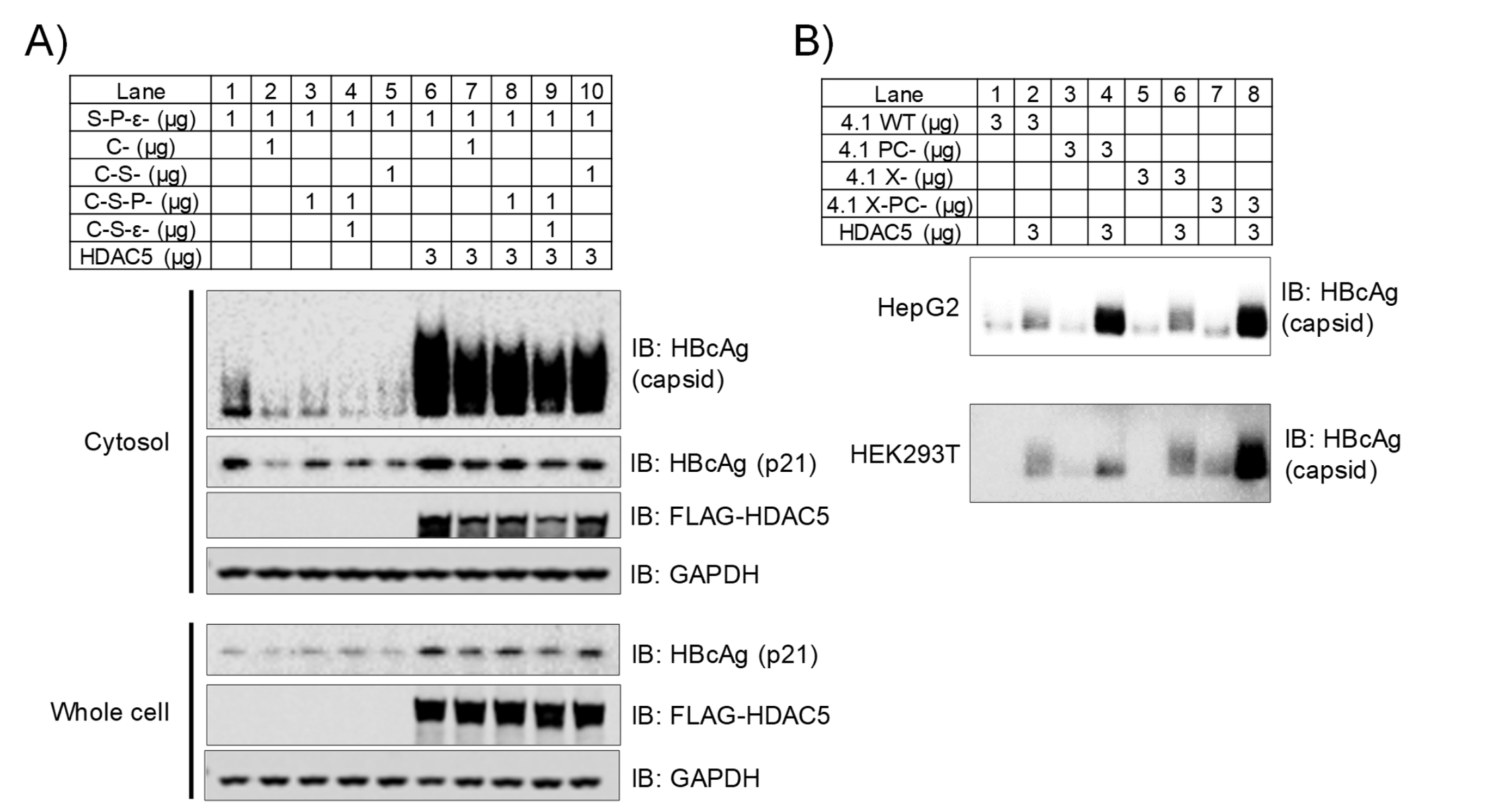

Supplement: S2 Fig — (A) HepG2 cells were transfected with the pCMVHBV construct lacking expression of the polymerase and surface proteins as well as the packaging signal ε (S-P-ε-). This construct was complemented with pCMVHBV constructs that lack expression of the core polypeptide but express the polymerase polypeptide (C-S- and C-S-ε-) or the polymerase and surface polypeptides (C-), in the presence or absence of the HDAC5 expression vector. The viral proteins were analyzed from cytoplasmic or whole cell extracts by Western blot analysis for native viral capsids and core polypeptide (p21). (B) HepG2 or HEK293T cells were transfected with the HBV DNA (4.1 kbp) construct or mutant constructs that lack production of the precore (4.1 PC-), X (4.1 X-), or both proteins (4.1 X-PC-) with or without the HDAC5 expression vector. The viral proteins were collected from the cytoplasm and examined by Western blot analysis for native viral capsids. (TIF) [file ppat.1008802.s002.tif]

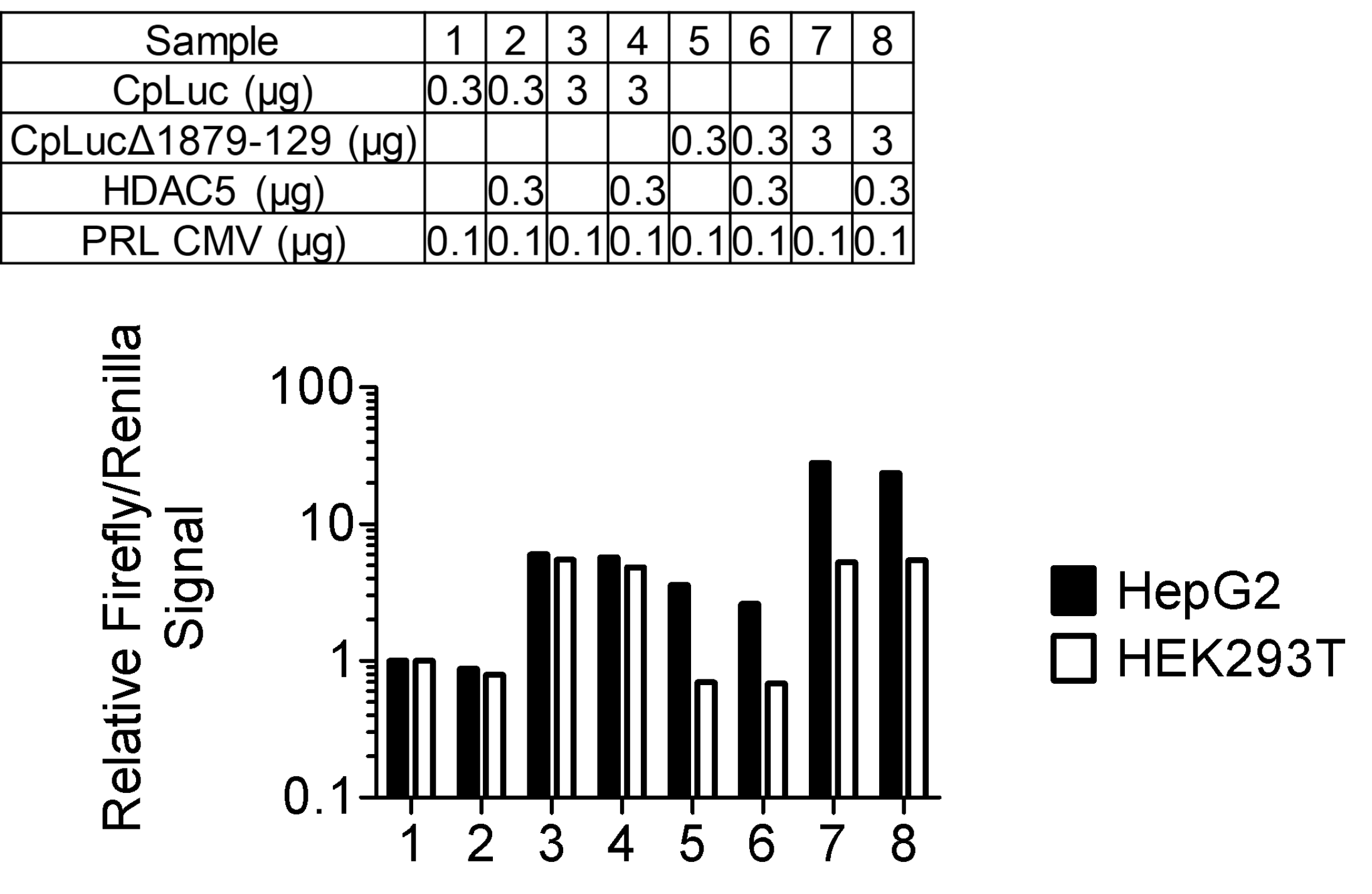

Supplement: S3 Fig — HepG2 and HEK293T cells were transfected with the HBV core promoter firefly luciferase reporter gene (CpLuc and CpLucΔ1879–129) and the CMV-driven Renilla luciferase reporter gene (pRLCMV) constructs [70]. HBV core promoter activity was normalized to Renilla luciferase activity and reported relative to the signal obtained in the cells transfected with 0.3 μg of the CpLuc construct and 0.1 μg of the pRLCMV construct which is designated as a relative activity of 1.0. (TIF) [file ppat.1008802.s003.tif]
