# Supplementary material for: Modulation of hepatitis B virus pregenomic RNA stability and splicing by histone deacetylase 5 enhances viral biosynthesis
Source: PLoS Pathog. 2020 Aug 21;16(8):e1008802. doi: 10.1371/journal.ppat.1008802 (PMC7467325; doi:10.1371/journal.ppat.1008802)
Supplement: Attachment — Submitted filename: PPATHOGENS-D-20-00724_Responsetoreviewers_AMcL.docx [file ppat.1008802.s005.docx]

June 4, 2020

Haitao Guo, Guest Editor

Guangxiang Luo, Section Editor

Kasturi Haldar, Editor-in-Chief

Michael Malim, Editor-in-Chief

PLOS Pathogens

Dear Editors,

Thank you for your e-mail of May 20, 2020, regarding our manuscript “Modulation of Hepatitis B Virus Pregenomic RNA Stability and Splicing by Histone Deacetylase 5 Enhances Viral Biosynthesis” by Taha et al. (PPATHOGENS-D-20-00724). We have modified the manuscript in view of the reviewers’ comments.

We appreciate the reviewers’ comments, and it is gratifying that “the reviewers considered that the study is novel and interesting.” Overall, it appears that the reviewers found the study provided insights not only into HBV post-transcriptional regulation but also into HDAC5 cellular functions. The reviewers’ concerns appear to stem from two primary issues: The use of specific cell lines in the study and the apparent discordance between the levels of HBV core polypeptide/p21 and viral capsids. We shall first try to address these primary issues.

(1) The human differentiated hepatoma cell replication system has permitted the molecular characterization of the various steps in the viral replication cycle. However, the use of these cells does not permit the simple identification of the individual host factors governing HBV biosynthesis. To address this, in 2001, we developed a novel HBV biosynthesis system utilizing the expression of exogenous liver-enriched transcription factors in nonhepatoma cells. This system revealed the critical importance of nuclear receptors, HNF4α, RXRα plus PPARα, RXRα plus FXRα, LRH1, and ERR, for HBV pregenomic RNA synthesis and viral DNA biosynthesis, which had not been apparent from studies utilizing human differentiated hepatoma cells. Indeed, this system permits the functionality of individual liver-enriched transcription factors to be evaluated independently of each other (or in defined combinations) in a manner that is not possible in human differentiated hepatoma cells. Using the nonhepatoma cell system, we demonstrate in the current study that HBV pregenomic 3.5 kb RNA can be stabilized by the expression of HDAC5 resulting in HBV biosynthesis in HEK293T cells. Similar, but more modest effects were observed in the human hepatoma cell line, HepG2 (where exogenous HDAC5 expression is more limited (see new data, Fig. S1C), likely accounting for the more modest effect observed on viral biosynthesis). These studies indicated that HBV RNA metabolism was directly regulated by post-transcriptional modifications of cellular targets governing viral biosynthesis. Despite the unique utility of the nonhepatoma cell system to model viral replication in hepatoma cells, a central concern regarding this system is the in vivo relevance. For this reason, we always attempt to confirm our findings from cell culture in vivo using the HBV transgenic mouse model of chronic HBV infection. We are currently in the process of generating Hdac5-null HBV transgenic mice to determine the in vivo role of HDAC5 in governing HBV RNA metabolism and viral biosynthesis (although this is beyond the scope of the current study). An interesting aspect of the HBV transgenic mouse model of chronic viral infection is the observation that HBV transcription and replication occurs at the highest levels in the hepatocytes adjacent to the central vein (perivenous hepatocytes) within zone 3 of the liver lobule, a pattern of expression which strongly correlates with HDAC5 expression across the liver lobule (see Figure S1B). These observations are consistent with our hypothesis that HDAC5 has a role in governing HBV biosynthesis in vivo. This is a unique and innovative approach which we alone have used to validate our transcriptional and post-transcriptional analysis by comparing both cell culture and mouse model systems of HBV biosynthesis.

(2) We have previously demonstrated (Ref. 37) that a modest increase in viral RNA is associated with a similar modest increase in HBcAg/p21 polypeptide which, nevertheless, is associated with a large increase in viral capsid synthesis and associated replication intermediates. This implies that viral capsid assembly is non-linear, cooperative, and concentration dependent, a phenomenon previously observed and rigorously investigated for HBcAg/p21 polypeptide alone in the absence of viral replication and now referenced within the manuscript (Refs 35, 36) on page 8 line 134. Our current study is in line with these previous observations that capsid assembly and viral biosynthesis occur in a cooperative fashion at least under the specific conditions observed in HEK293 cells, providing further validation for the common nature of this phenomenon.

Our specific responses to the reviewers’ comments are included below and modifications to the manuscript can be identified by reviewing the track changes.

**Part I – Summary**

Reviewer 1 states “*Considering that the phenotype from HepG2 cells (Fig. 1A) was not so impressive, and that no work in Huh7 cells was described, the physiological relevance of the findings from HEK293T cells is somewhat questionable. Moreover, since HEK293T is not a good system to recapitulate late steps of HBV lifecycle such as genome replication, the HBV parameters observed could be somewhat different from well established Huh7 and HepG2 cells due to missing liver specific co-factors.*”

We agree with the reviewer that the effect in HepG2 cells is not as impressive compared with the effect in HEK293T cells. This could be due to the relative abundance of HDAC5 in these two cell lines upon transfection with the HDAC5 expression vector. HepG2 cells display lower expression of the exogenous HDAC5 protein compared with HEK293T cells. The reason for this difference is currently unclear but it may indicate that HepG2 cells are more representative of the periportal environment of the liver lobule while HEK293T cells are more representative of the pericentral environment, at least with regard to HDAC5 protein expression and/or stability. This data is now provided in Fig. S1C. Additionally, it is also mentioned in the text as “Moreover, the limited expression of HDAC5 protein in HepG2 cells compared with HEK293T cells (Supplementary Fig. S1C) may explain the modest effect of HDAC5 expression on HBV biosynthesis in HepG2 cells compared with the robust effect in HEK293T cells.” on page 8 line 134-137. Collectively, the qualitative effect of HDAC5 expression can be observed in both cell lines and its effect on intracellular viral DNA correlates with the relative level of HDAC5 expression which strongly indicates the biological importance of HDAC5 in the post-transcriptional regulation of HBV biosynthesis.

Reviewers 2 and 3 only made positive comments regarding the study in this part of the review and did not have comments/concerns that need to be addressed.

**Part II – Major Issues: Key Experiments Required for Acceptance**

Reviewer 1, point 1, part 1. The reviewer states “*The most striking finding from Fig. 1 is the discordance between core protein by Western blot vs. core particles by native agarose gel, in both HepG2 cells and 293T cells (according to P8, HDAC5 increased core protein by 5 fold but core particles by 50 fold in 293T cells). Such discordance was observed throughout this study, but no explanation was provided.*”

We agree with the reviewer that an explanation of this apparent discordance is warranted. It has previously been reported that a modest increase in HBcAg/p21 polypeptide could be associated with a large increase in viral capsid synthesis and associated replication intermediates. This implies that viral capsid assembly is cooperative and concentration dependent as we and others have previously noted. A comment to this effect is now included in the text as “The observation that an HDAC5-mediated 5-fold increase in HBV core polypeptide (p21) levels is associated with a 50-fold enhancement in HBV capsid abundance is consistent with previous reports indicating that the conversion of HBcAg dimers into capsids occurs by a highly cooperative process (35-37)” on page 8 line 130-134.

Reviewer 1, point 1, part 2. The reviewer states “*Did the deletion of last residue from core protein dramatically enhance core particle assembly (either as a mixture with wild-type core protein or by itself)?*”

This is an excellent question regarding the implications of deleting the terminal cysteine residue in HBcAg. There is an apparent slight, 1.5-fold, enhancement in capsid assembly and viral replication when the truncated core polypeptide was expressed. We had addressed this question with the data shown in Fig. S4. This figure is now moved to the main manuscript given that its importance was rightfully highlighted by the reviewer. Quantification of the abundance of capsids and replication intermediates is included for clarity. It suggests that there is a very modest increase in capsid assembly and viral replication with the truncated core polypeptide but a more prominent enhancement of capsid assembly when wild type and truncated HBV core polypeptides are co-expressed. This is also addressed in the text as “This truncated HBV core polypeptide appears to support capsid assembly and viral replication slightly more efficiently than the wild type HBV core polypeptide (Fig. 6). Additionally, modulating the ratio of the wild type to truncated HBV core polypeptide appears to increase capsid assembly to a modest extent without affecting viral replication (Fig. 6).” on page 13 line 242-248.

Reviewer 1, point 1, part 3. The reviewer states “*did the loss of a cysteine markedly enhance binding of the core particles to the antibody used for detection? Has the authors tried a different anti-HBc antibody?*”

Generally, antibodies recognizing native HBV capsids bind to the spike region of the core protein and therefore deletion of the cysteine residue should not affect antibody binding. This is supported by the fact that we did not observe differences in sensitivity when using different core protein antibodies. The antibody used in these studies specifically recognizes amino acids 74-80 in the spike region of the core polypeptide sequence (Santa Cruz Biotechnology, antibody “HBcAg (1-5)” as noted on page 22, line 429).

Reviewer 1, point 2, part 1. The reviewer states “*Many previous studies focused on the 2.2-kb spliced pgRNA, which were cited by the authors. Did the other researchers find more efficient core particle formation or discordance between core protein and core particles similar to the current work?*”

The reviewer is correct that many have studied the 2.2 kb splice variant of the 3.5 kb RNA, but those studies (Refs 6, 11-13, 16) did not investigate HBV capsid assembly from the truncated HBV core polypeptide.

Reviewer 1, point 2, part 2. The reviewer states “*can the 2.2-kb spliced RNA be packaged into core particles and be converted to rcDNA? From the Southern blot shown in Figs. 1 and 2, HDAC5 did not alter the size of replicative DNA.*”

The 2.2 kb spliced RNA contains the ε packaging signal and it is packaged into capsids; it has been detected in sera of infected patients, HBV transgenic mouse liver, and in transfected cell lines. This splice variant is reverse transcribed to DL DNA but has not been shown to be converted to RC DNA (Ref 13). This was mentioned in the Discussion as “this transcript does not encode a functional viral reverse transcriptase/DNA polymerase and cannot be reverse transcribed to generate relaxed circular HBV 3.2 kb DNA (13).” on page 15 line 280-282. We agree that we did not observe an alteration to the HBV DNA RI profile in the Southern blot when HDAC5 was expressed. This is likely due to the complex nature of the RI profile and the necessity to develop appropriate assays to specifically detect RIs derived from the spliced HBV transcript. This was not attempted as part of this study as it had already been described by others (Ref 13). However, as noted above the HBV 2.2 kb spliced RNA does not encode a functional RT/pol and hence will be packaged with wild-type RT/pol in trans, a highly inefficient process. Consequently, the HBV 2.2 kb RNA would only be expected to contribute to the viral DNA replication intermediates to a very limited extent. This explains why increased splicing does not lead to an observable effect on the intracellular DNA replication intermediate profile.

Reviewer 1, point 3, part 1. The reviewer states “*In Fig. 1B, top panel, the Northern blot pattern is rather complex. There are two high molecular weight bands at where 3.5-kb was marked, and between that and the marked 2.1-kb RNA there is another band. Moreover, the 0.7-kb RNA is quite strong relative to HepG2 cells. For the lowest panel in Fig. 1B, which top band was used as 3.5-kb RNA?*”

We agree with the reviewer that the Northern blot HBV transcript profile is complex, which is due to the many viral transcripts and their associated splicing products. The second major band from the top represents the HBV 3.5 kb band, while the top band probably represents the previously reported HBV long xRNA that initiates from the X promoter region (Ref 76). This was mentioned in the figure legend of each figure where this band was observed. Additionally, this band is absent when viral biosynthesis from pregenomic RNA is driven by the CMV promoter rather than the HBV nucleocapsid promoter (pCMVHBV construct, Fig. 3C), further supporting the conclusion that the HBV 3.9 kb RNA is the HBV long xRNA described previously. Additionally, the band that is between the top two bands and the 2.1 kb band has not yet been characterized but might be the singly spliced product (2.7 kb) of the long xRNA that initiates from the X promoter region. The higher abundance of the 0.7 kb RNA in HEK293T cells compared with HepG2 cells is consistent with the previous studies in these cell lines (Ref 37). To address this complexity, we have now included arrows on the Northern blot to indicate all the RNA bands that were labeled and used for quantification. Collectively, we believe that measurement of transcript abundance by three methods (Northern blot, RT-qPCR, and primer extension) has provided us with an accurate representation of the effect of HDAC5 expression on viral transcript splicing and stability.

Reviewer 1, point 3, part 2. The reviewer states “*the Southern blot image showed marked increase of replicative DNA in lane 5 similar to increase in capsids, but according to quantification at the bottom, DNA replication increased by less than 6 fold whereas capsids increased by 60 fold*.”

We agree with the reviewer that the increase in HBV DNA RI may be more robust than 6-fold. Quantification of lane 1 of the Southern blot where the RI signal is very modest and background estimation is more challenging could be responsible for part of the estimated discordance. The cooperative assembly of empty as well as nucleic acid-containing HBV capsids may also contribute to greater increases in total capsid abundance compared with a more modest enhancement in observed DNA RIs.

Reviewer 1, point 4, part 1. The reviewer states “*According to P9, line 160-162, co-transfection with 10ug of HDAC5 WT or its H1006Y mutant, there is 2-fold increase in 2.1-kb RNA. Visual inspection of Fig. 2B suggested more than 2-fold increase by the H1006Y mutant. But the increase in core particles for this mutant was nearly 1000 fold according to the bottom panel! Is such a number reliable?*”

This question is addressed by the answer to reviewer 1, point 1, part 1 above. These results are in line with previous reports that modest increase in HBV RNA and therefore modest increase in HBcAg/p21 polypeptide could be associated with a large increase in viral capsid synthesis and associated replication intermediates. This implies that viral capsid assembly is cooperative and concentration dependent. Given that the comparison is based on lane 1 where capsids are barely detectable, it is not unreasonable that a major enhancement of capsid assembly by the HDAC5 H1006Y mutant will lead to the calculated 700-fold increase reported.

Reviewer 1, point 4, part 2. The reviewer states “*If so why did not the authors use the H1006Y mutant to clarify the underlying mechanism in the subsequent figures?*”

Class IIa HDACs have relatively low enzymatic activity when compared to other HDAC isoforms. Therefore, in addition to utilizing the small molecule inhibitor LMK235 to address the requirement of HDAC5 catalytic activity for the effect on HBV biosynthesis, we constructed the HDAC5 high catalytic activity mutant H1006Y. This mutant enables investigation of the role of HDAC5 enzymatic activity on HBV biosynthesis without the use of small molecule inhibitors and their associated potential off-target effects. The use of the construct demonstrated that, indeed, upregulation of HDAC5 catalytic activity using the H1006Y mutant was associated with an upregulation of HBV biosynthesis. Given that the wild-type HDAC5 enzyme has a robust effect on HBV biosynthesis, we aimed to elucidate the mechanism in subsequent studies utilizing the wild-type HDAC5 construct. It is not apparent to us how utilizing the H1006Y HDAC5 mutant instead would clarify the mechanism better.

Reviewer 1, point 5. The reviewer states “*According to Fig. 3C, in Northern blot there is little change in 3.5-kb RNA but marked increase in 2.2-kb RNA. According to Fig. 5B (qPCR) (text in P12, lines 233-235), HDAC5 increased 3.5-kb RNA by 3 fold, and 2.2-kb RNA by 33 fold). Such a finding could explain the increase in both core protein and capsids, but not the much dramatic increase in capsids than core protein.*”

We agree with the reviewer that both the Northern blot in Fig. 3C and the RT-qPCR data in Fig. 5B are consistent with a modest effect of HDAC5 on 3.5 kb RNA levels. It is the dramatic increase in the HBV 2.2 kb spliced RNA that is critical. Together, the quantification of the viral transcripts by three different methods including Northern blot, primer extension and RT-qPCR indicate that HDAC5 increases HBV 3.5 kb RNA stability and splicing to the 2.2 kb RNA. As discussed above for reviewer 1, point 1, part 1, the cooperative assembly of HBV capsids explains the modest increases in HBV core polypeptide but major enhancement in HBV capsid abundance.

Reviewer 1, point 6. The reviewer states “*Fig. 6 was meant to show prolonged half-lives of both 3.5-kb and 2.2-kb RNAs in the presence of HDAC5. But why was the 3.5-kb RNA much less abundant when HDAC5 was present at time point “0”?*”

Regarding the data presented in figure 7 (previously figure 6), the levels of the HBV transcripts were normalized to the levels of the GAPDH RNA. The GAPDH RNA is indeed slightly lower in the “zero” timepoint when HDAC5 is expressed compared with the “zero” timepoint when HDAC5 is not expressed. Therefore, this is likely a loading issue rather than a reduction in viral RNA. Critically, actinomycin D was used to inhibit transcription at 40 hours post-transfection (not the typical 72 hours) so RNA decay could be estimated over the subsequent 24 hours. Estimates of RNA decay were determined relative to the “zero” time point for each condition (plus and minus HDAC5 expression) to evaluate the effect of HDAC5 on viral RNA stability. This analysis clearly demonstrated that HDAC5 increased HBV RNA stability at each time point examined.

Reviewer 2, point 1. The reviewer states “*A central finding of the manuscript is that HDAC5 expression both increases the stability of the 3.5 kb HBV transcript and promotes the splicing of this transcript to the 2.2 kb form. However, this seems counterintuitive. If 3.5 kb RNA splicing to 2.2 kb is increased, it would seem that the half-life of the unspliced 3.5 kb transcript might be decreased, rather than increased (Fig 6), at least in the nucleus. This might be understandable if only cytoplasmic RNA were being analyzed, but the methods state that total RNA was used, and the model in Fig 7 appears to imply different relative amounts of the transcripts in the nucleus.*”

We appreciate the reviewer’s comment regarding the issue of relating RNA splicing to stability. We have addressed this in the Discussion as “It is generally considered that one of the major non-coding functions of RNA splicing is to increase RNA stability although the precise mechanisms may depend on a variety of distinct pathways (46)” on page 15, lines 288-290. Given that the splicing factors/RNA binding proteins must recognize and bind to HBV RNA for splicing to occur, this appears to result in a general increase in viral transcript stability. Additionally, this binding is associated with the splicing of a fraction of the HBV 3.5 kb RNA to generate the HBV 2.2 kb spliced transcript. Overall, the action of HDAC5 leads to an increase in the total steady state HBV 3.5 kb RNA due to increased stability which more than compensates for the loss due to splicing, with associated dramatic increase in the HBV 2.2 kb spliced RNA.

Reviewer 2, point 2. The reviewer states “*Although HDAC5 overexpression is clearly affecting HBV RNA splicing and capsid formation, the cellular mechanism of this effect is not defined. As the authors speculate, it could be due to the direct regulation of the splicing machinery by HDAC5. Alternatively, it could be an indirect effect of HDAC5 on the expression of other cellular genes. Furthermore, the results section ends somewhat abruptly with the implication that expression of the truncated Core variant from the 2.2 kb transcript is driving increased HBV capsid assembly, but this could have been further explored. Although discussed, the lack of mechanistic details is nevertheless a weakness of the study.*”

We agree with the reviewer that the mechanistic details of the effect of HDAC5 on HBV biosynthesis are not elucidated in the current study. We are in the process of addressing it. These studies are beyond the scope of the current manuscript.

Reviewer 2, point 3. The reviewer states “*The experiments are mostly all carried out using transformed cell lines (HepG2 and HEK293T) that likely have differences in HDAC expression, histone acetylation, and RNA splicing compared to normal cells. The mouse liver data shown in Figure S1 are exciting but also indirect. Figure S1B shows apparent differences in HDAC5 expression across liver zones by single-cell RNA-Seq. A similar single-cell analysis could be performed using HBV transgenic mice to show a direct relationship between HDAC5 expression and HBV RNA expression and splicing in the liver.*”

We agree with the reviewer that single-cell analysis of HBV transgenic mouse liver would permit the correlation of HDAC5 expression and HBV RNA synthesis and splicing across the liver lobule. However, this is a major undertaking beyond the scope of the current analysis. Furthermore, we are planning to generate and characterize the Hdac5-null HBV transgenic mouse to directly determine the role of HDAC5 in governing HBV biosynthesis in vivo.

Reviewer 3, point 1. The reviewer states “*Fig. 1B - While there is no doubt that HDAC5 increased HBV DNA RI, the quantification provided seems inconsistent with the magnitude of change in the figure. The levels are clearly much more increased than the 3- and 6-fold indicated. I would ask the authors to check this and perhaps consider assessing the DNA levels more quantitatively via qPCR.*”

This issue is addressed above in response to reviewer 1, point 3, part 2 with regards to quantification of the Southern blot. We have considered (and attempted) using qPCR to assess the levels of DNA RI more quantitatively. However, the results are unreliable given that these are transient transfection experiments that lead to the contamination of HBV RIs with small fragments of plasmid DNA despite extensive DNase digestion of cytoplasmic capsid preparations.

Reviewer 3, point 2. The reviewer states “*The use of the term epigenetics does not seem appropriate to the effect being reported here. My albeit limited understanding is that epigenetic changes are by definition inheritable. Thus, it does not appear that this specific post-translational modification would fall under the category of an epigenetic change.*”

We completely agree with the reviewer that the term epigenetic may not be appropriate here as there is no evidence for inheritance of the effect of HDAC5 on HBV biosynthesis. We have replaced the word “epigenetic” throughout the manuscript with “post-transcriptional” where appropriate.

**Part III – Minor Issues: Editorial and Data Presentation Modifications**

Reviewer 1 did not have any comments in this section.

Reviewer 2, point 1. The reviewer states “*The HDAC5 inhibitor LMK235 only had an effect at the highest concentration (250 nM), and it is not clear that the drug is specific for HDAC5 at this concentration (Fig 2A). Biochemical analysis of specificity or confirmation by RNAi knockdown is necessary to interpret this result. Similarly, Fig 2B would be strengthened by a demonstration of increased HDAC5 activity in the HDAC5 mutant-transfected cells.*”

The effect of LMK235 is to inhibit HDAC5-dependent HBV biosynthesis which argues strongly it is directly acting on HDAC5, a known target molecule. We agree with the reviewer that it is often difficult to interpret results obtained using small molecule inhibitors (due to selectivity issues) and this is the reason we independently confirmed the necessity for HDAC5 catalytic activity utilizing an orthogonal approach - the high activity mutant H1006Y in figure 2B. The activity of the H1006Y mutant has been previously demonstrated (Ref 39) to be 50-fold higher than wild-type HDAC5. Regardless, biochemical analysis of LMK235 HDAC selectivity in vitro (Ref 38) demonstrated that it is selective for HDAC5, or at least class IIa HDACs, at or below a concentration of 300 nM. In addition, biochemical IC_50_ values typically obtained in vitro are generated using synthetic substrates selected based on the magnitude of the readout effect. These synthetic substrates, although useful for guiding design of novel inhibitors, are poor representation of the multitude of the substrates encountered endogenously. Other phenomena, such as compound degradation in culture media and cells, non-specific binding, and cell permeability can further complicate the choice of the concentration for use in cell culture. For this reason, the concentration that is functional in cells is often thought to be ca. 10-fold higher than the concentration obtained in vitro. Taking these considerations into account, we anticipate that LMK235 is HDAC5, or at least class IIa HDAC, selective at the concentrations used in this study. In conjunction with the data using the high activity mutant H1006Y, the data obtained with LMK235 clearly demonstrate that HDAC5 catalytic activity upregulates HBV biosynthesis.

Reviewer 2, point 2. The reviewer states “*Steps 8 and 9 in the model (Figure 7) are not directly supported by any data in the manuscript and should be removed.*”

We agree with the reviewer that steps 8 and 9 in the model are not supported directly by our study but they are well established steps in HDAC5 nuclear localization, and we believe that it is necessary to keep these steps in the model for completeness. Further investigation of steps 8 and 9 in relation to HBV biosynthesis is certainly warranted in future studies. Importantly, these steps are potentially relevant to the post-transcriptional cytokine modulation of HBV biosynthesis as noted by reviewer 3 below.

Reviewer 3, point 1. The reviewer states “*Lines 163-164 – The authors state the data showing an enhanced effect with the HDAC5 mutation with enhanced enzymatic activity “indicate that HDAC5-mediated support of HBV biosynthesis requires the enzymatic activity of HDAC5”. However, technically this mutant does not show the enzymatic activity is REQUIRED (for that you would need an enzymatically dead mutation), it rather simply shows a correlation with the enzymatic activity. I would recommend a slight re-phasing to avoid overstatement.*”

We agree with the reviewer that the data provided does not show the requirement for catalytic activity, but it rather demonstrates that HDAC5 catalytic activity is associated with an increase in HBV biosynthesis. To that end, we have changed the title of this section of the results to “HDAC5 catalytic activity upregulates HBV biosynthesis” on page 8, line 138. We have also re-phrased the last sentence in the section to “These observations indicate that the catalytic function of HDAC5 contributes to enhanced HBV biosynthesis.” on page 9, lines 165-167.

Reviewer 3, point 2, part 1. The reviewer states “*The implications of this work seem rather far reaching in several areas which could be discussed to a greater extent in the discussion. Obviously, this is not a requirement for publication but a suggestion to the authors. For example: - There is quite an extensive literature on immune mediate post-transcriptional regulation of HBV. Could this be a major mechanism behind these observations? If so, such a link to the pre-existing literature would be useful to discuss.*”

We agree with the reviewer regarding the immune mediated post-transcriptional regulation of HBV biosynthesis, such as inhibition of vial biosynthesis with proinflammatory signals (e.g. poly I:C treatment/interferon α/β induction). Indeed, these signals have been shown to dramatically decrease viral replication with very modest effects on transcription. Inhibition of the effect of HDAC5 using these immunological stimuli would inhibit splicing and generation of the core polypeptide-encoding 2.2 kb RNA while also destabilizing the 3.5 kb RNA which together would decrease the overall levels of core protein and therefore inhibit replication. We have now addressed this possibility in the Discussion by adding the following “Indeed, these kinases are activated by antiviral factors such as nucleos(t)ide analogs therapeutics (54) and proinflammatory signals, including interferon (55), poly I:C (56), lipopolysaccharide (56, 57) and tumor necrosis factor alpha (TNFα) (57). This potentially suggests a role of HDAC5 in mediating the inhibitory effects of these antiviral factors. Activation of CaMK and AMPK by these stimuli leads to the nuclear export of phopho-HDAC5 and would presumably eliminate its interaction with relevant host factors (48), resulting in reduced splicing and stability of the HBV 3.5 kb RNA and thereby down regulating viral biosynthesis. This might be reflected as a modest decrease in HBV 3.5 and 2.2 kb RNA levels, which potentially could be missed without the appropriate analysis to distinguish between the HBV spliced 2.2 kb and unspliced 2.1 kb RNAs. However, this change in viral transcript profile might result in a modest reduction in core/p21 polypeptide with an associated dramatic loss of capsid formation due to the concentration-dependent cooperative process involved in capsid assembly (35-37). In fact, studies utilizing proinflammatory signals typically report a post-transcriptional inhibitory effect on HBV biosynthesis associated with limited changes in viral RNA levels but with a loss of cytoplasmic capsids (58, 59).” on page 17 lines 316-334. The relationships among proinflammatory signals, HBV biosynthesis and HDAC5 warrant future investigation but are beyond the scope of the current study.

Reviewer 3, point 2, part 2. The reviewer states “*How much of the “2.1kb HBV RNA” observed on NB in the literature is actually this 2.2kb spliced core transcript and how might that have impacted led to the misinterpretation of HBV NBs over the years when changes in the NB band were presumed to reflect the level of the 2.1kb HBs transcript*”

We agree with the reviewer that this is a critical aspect of analyzing HBV RNAs using Northern blotting and we have now included this in the Discussion section as part of addressing the previous issue (Reviewer 3, point 2, part 1).

I hope my responses to the reviewers' comments are satisfactory. Thank you for your help in this matter.

Yours sincerely

Alan McLachlan, Ph.D.,

Professor

Department of Microbiology and Immunology (MC 790)

College of Medicine

University of Illinois at Chicago

Room 8091, 909 South Wolcott Avenue

Chicago, IL60612-7344

Phone: (312)355-0211

Fax: (312)996-4890

E-mail: [mclach@uic.edu](mailto:mclach@uic.edu)
